# Effects of Glutathione Diminishment on the Immune Responses against *Mycobacterium tuberculosis* Infection

**DOI:** 10.3390/app11178274

**Published:** 2021-09-06

**Authors:** Ruoqiong Cao, Afsal Kolloli, Ranjeet Kumar, James Owens, Kayvan Sasaninia, Charles Vaughn, Mohkam Singh, Edward Truong, Nala Kachour, Abrianna Beever, Wael Khamas, Selvakumar Subbian, Vishwanath Venketaraman

**Affiliations:** 1 Department of Basic Medical Sciences, College of Osteopathic Medicine of the Pacific, Western University of Health Sciences, Pomona, CA 91766, USA;; 2 Public Health Research Center, New Jersey Medical School, Rutgers University, Newark, NJ 07103, USA;; 3 Graduate College of Biomedical Sciences, Western University of Health Sciences, Pomona, CA 91766, USA;; 4 College of Veterinary Medicine, Western University of Health Sciences, Pomona, CA 91766, USA;

**Keywords:** tuberculosis, glutathione, cytokines, oxidative stress, inflammation

## Abstract

*Mycobacterium tuberculosis (M. tb)*, the causative agent of tuberculosis (TB), continues to be a global health burden. We have reported that patients with marked deficiency in the production of glutathione (GSH) had impaired granulomatous effector responses against *M. tb* infection, which were restored when supplementing patients with liposomal GSH (lGSH). However, the effects of GSH deficiency in the lung parenchyma in altering granuloma formation and effector responses against *M. tb* infection remain unexplored. We aim to elucidate the effects of diethyl maleate (DEM)-induced GSH deficiency during an active *M. tb* infection in an in vivo mouse model. We assessed for total and reduced GSH levels, malondialdehyde (MDA) levels, cytokine profiles, granuloma formation and *M. tb* burden. DEM administration significantly diminished total and reduced GSH levels in the lungs and plasma and increased MDA levels in infected mice compared to sham-treated controls. DEM treatment was also associated with an increase in IL-6, TNF-α and ill-formed granulomas in infected mice. Furthermore, *M. tb* survival was significantly increased along with a higher pulmonary and extrapulmonary bacterial load following DEM treatment. Overall, GSH deficiency led to increased oxidative stress, impaired granuloma response, and increased *M. tb* survival in infected mice. These findings can provide insight into how GSH deficiency can interfere with the control of *M. tb* infection and avenues for novel therapeutic approaches.

## 1. Introduction

Tuberculosis (TB) is one of the oldest documented infectious diseases in the world. The etiological agent, *Mycobacterium tuberculosis* (*M. tb*), is responsible for causing about 1.2 million deaths and 10 million cases of active disease annually, worldwide [1]. The pathogen is transmitted through inhalation of infectious aerosols, which can allow *M. tb* to enter the alveoli in the lungs [2]. As an innate immune response to infection, *M. tb* is internalized by alveolar macrophages via receptor-mediated phagocytosis. However, virulence factors of *M. tb* prevent phagocytic degradation of the bacteria and ensure its survival in the macrophage, leading to a primary infection [3]. Infected phagocytes migrate into the lung parenchyma and pulmonary lymph nodes, mounting a Th-1 cytokine response that recruits various immune cells to create a granuloma at the site of infection [4]. The granuloma creates a physical barrier and a hypoxic environment that deprives *M. tb* of nutrients required for its replication, thus suppressing active infection, and localizing the pathogen to the lungs [5,6]. If the pathogen is not eliminated, *M. tb* persists in a quiescent state in the granuloma, leading to latent tuberculosis infection (LTBI) [7–11].

Glutathione (GSH) is an endogenous antioxidant tripeptide composed of glutamate, cysteine, and glycine. The two forms of GSH are reduced GSH (rGSH) and oxidized GSH (GSSG). rGSH is the functional form, containing antioxidant characteristics which prevent cellular damage from reactive oxygen species (ROS). rGSH reacts with ROS, producing GSSG and water, mitigating the ROS and any corresponding potential damage [12,13]. We have previously reported that GSH is directly toxic to *M. tb* and can serve as an important effector molecule mediating macrophage control of *M. tb* [14]. Furthermore, GSH augments the function of natural killer (NK) cells and has direct antimycobacterial activity, thus enhancing immune effects [9,10]. Immunocompromised individuals, such as those infected with HIV, appear to have decreased GSH levels and thus a compromised defense mechanism against the growth of *M. tb* [9]. However, our previous studies suggest that restoration of GSH in HIV-positive subjects could potentially result in redox homeostasis, cytokine balance and improved immune responses against *M. tb* infection [11,12].

Patients with T2DM exhibit diminished synthesis of GSH-synthesizing enzymes, including the catalytic subunit of glutamine-cysteine ligase (GCLC), and thus produce lower levels of total GSH [9,13]. T2DM patients also exhibit increased production of malondialdehyde, a byproduct of lipid peroxidation and a marker for oxidative stress [15,16]. Patients with T2DM also have an increase in proinflammatory cytokines and an inadequate Th-1 effector response [16]. Peripheral blood mononuclear cells (PBMCs) isolated from T2DM patients showed an impaired ability to form granulomas in vitro when infected with Erdman strain *M. tb* compared to isolates derived from healthy patients [15,17]. In addition, in vitro *M. tb* survival and growth in infected PBMCs from T2DM patients is significantly increased compared to healthy controls [17]. Similar to previous studies performed with HIV-positive patients, supplementation with a liposomal formulation of GSH in T2DM subjects restored GSH levels and consequently decreased oxidative stress, modulated a Th-1 cytokine response to *M. tb*, and enhanced in vitro granuloma formation and *M. tb* control [8,17–19]. Altogether, these studies suggest that GSH fills important immunomodulatory roles in granuloma formation and in the control of *M. tb* growth in vitro.

However, the effects of GSH deficiency in the lung parenchyma and its impact on the innate and adaptive immune response during an active *M. tb* infection have yet to be explored. We therefore aim to develop an in vivo mouse model to determine the effects of GSH deficiency on the effector and granulomatous responses against *M. tb* infection in the lung parenchyma of C57BL/6 mice and explore cytokine production in the respiratory tract when GSH deficiency is induced. Diethyl maleate (DEM) is a known GSH-decreasing agent and was used to induce GSH-deficient conditions in *M. tb* infected mice [14]. GSH diminishment is achieved through enzymatic conjugation of DEM to GSH via a thioester bond, inactivating GSH activity [20,21]. The results of our in vivo studies indicate that DEM treatment exacerbated the inflammatory response, leading to a redox imbalance and thereby increasing the burden of *M. tb* in the lungs and spleen of the mice.

## 2. Materials and Methods

To evaluate the role of GSH in controlling *M. tb* infection and disease progression, mice were aerogenically infected with *M. tb* H37Rv and placed into either untreated or DEM-treated groups. Six mice from each group were sacrificed at specific time points: 2 weeks, 4 weeks, and 8 weeks post-infection, and samples were analyzed using various assays with hematoxylin and eosin staining, which were conducted for microscopic evaluation of tissue morphology.

### 2.1. Bacteria and Chemicals

*Mycobacterium tuberculosis* H37Rv (*M. tb*) strain was obtained from Dr. Thomas Shinnick (Center for Disease Control (CDC), Atlanta, GA, USA). The bacteria were grown to OD600 = 0.7 in Middlebrook 7H9 medium (Difco BD, Franklin Lakes, NJ, USA) supplemented with 10% ADC (albumin dextrose catalase) enrichment, aliquoted and stored frozen at −80 °C. To prepare inoculum for mice aerosol infection, stock vials were thawed and used as described in previous research [22].

### 2.2. Aerosol Infection, Treatment and CFU Assay

For animal studies, 8–10 weeks old C57BL/6 mice (n = 42; 21 male and 21 female mice) were purchased from the Jackson Laboratory (Bar Harbor, ME, USA). Bacterial stocks were generated as described previously [22]. Mice were exposed for 40 min to nebulized bacteria at a density optimized to deliver a standard low dose of around 50–100 CFU using Glas-Col Full Body Inhalation Exposure as described previously [23–28]. To determine the actual infection dose, six mice (3 male and 3 female) were sacrificed at three hours post-infection (3 h p.i.), lung homogenate was plated on Middlebrook 7H11 media (Difco BD, Franklin Lakes, NJ, USA) and CFU were counted after 4–6 weeks of incubation. After infection, mice were segregated into two groups: (1) untreated, and (2) DEM (diethylmaleate, Santa Cruz Biotechnology, TX, USA)-treated. Each group contained 18 mice (9 female and 9 male). DEM was administered daily (50 mM in 100 μL) by oral gavage for eight weeks. At specific time points (2-, 4- and 8-weeks post-infection), six mice (3 male and 3 female) from each group were euthanized and approximately 0.7 mL of blood was collected by cardiac puncture. A typical necropsy was performed, and lungs, spleens, and livers were harvested. The left lung and a portion of the spleen (25 mg) and liver (0.5 g) were homogenized in PBS with 0.05% tween 80. Homogenates were serially diluted and plated on Middlebrook 7H11 media. The plates were incubated at 37 °C and CFUs were counted after 4–6 weeks. The lower lobe of the right lung as well as a portion of spleen and liver were fixed in 10% neutral buffered formalin and used for histology studies. Plasma and lung lysates were filtered through a 0.2-micron filter and used for downstream works. All animal procedures were performed in bio-safety level 3 facilities as per the approved procedures of the Rutgers University Institutional Animal Care and Use Committee.

### 2.3. Histology Staining

Portions of lungs were fixed in 10% neutral formalin solution, paraffin-embedded and cut into 5 μm sections for staining with hematoxylin-eosin (H&E) to visualize the organization and distribution of leukocytes. The stained sections were analyzed using a Nikon Microphot DXM 1200C microscope and photographed using NIS-Elements software (Nikon Instruments Inc., Melville, NY, USA) as described previously [23]. Morphometric analysis was performed with the PathScan Enabler system (Meyer Instruments, Houston, TX, USA) and calculated using SigmaScan Pro software (Systat Softwares, San Jose, CA, USA) as described previously (31).

### 2.4. GSH, MDA, and Cytokine Measurements

In order to determine the effects of GSH depletion caused by DEM treatment, the levels of the total and reduced forms of GSH in the lung tissue homogenates and plasma from *M. tb*-infected mice were measured spectrophotometrically using an assay kit procured from Arbor Assays (Cat. # K006-H). Levels of IL-6 and TNF-α in the lung tissue homogenates and plasma from *M. tb*-infected mice were measured using enzyme-linked immunosorbent assay (ELISA) kits procured from Thermofisher (Cat. # BMS603-2 and BMS607-3). MDA levels in the lung tissue lysates and plasma were measured spectrophotometrically using an assay kit procured from Cayman Chemicals (Item # 10009055).

### 2.5. Statistical Analysis

GraphPad Prism Software 8 was utilized for statistical analysis. An unpaired t-test was performed when comparing only two categories with Welch corrections applied. All values reported represent the means for each respective category with *p*-values of <0.05 considered significant. Any placement of an asterisk (*) denotes a direct comparison to the previous category. When two asterisks are represented (**), a *p*-value below 0.005 is implied. When three asterisks are represented (***), a p-value below 0.0001 is implied.

## 3. Results

### 3.1. Assay of Total and Reduced Forms of GSH

**Figure 1.**
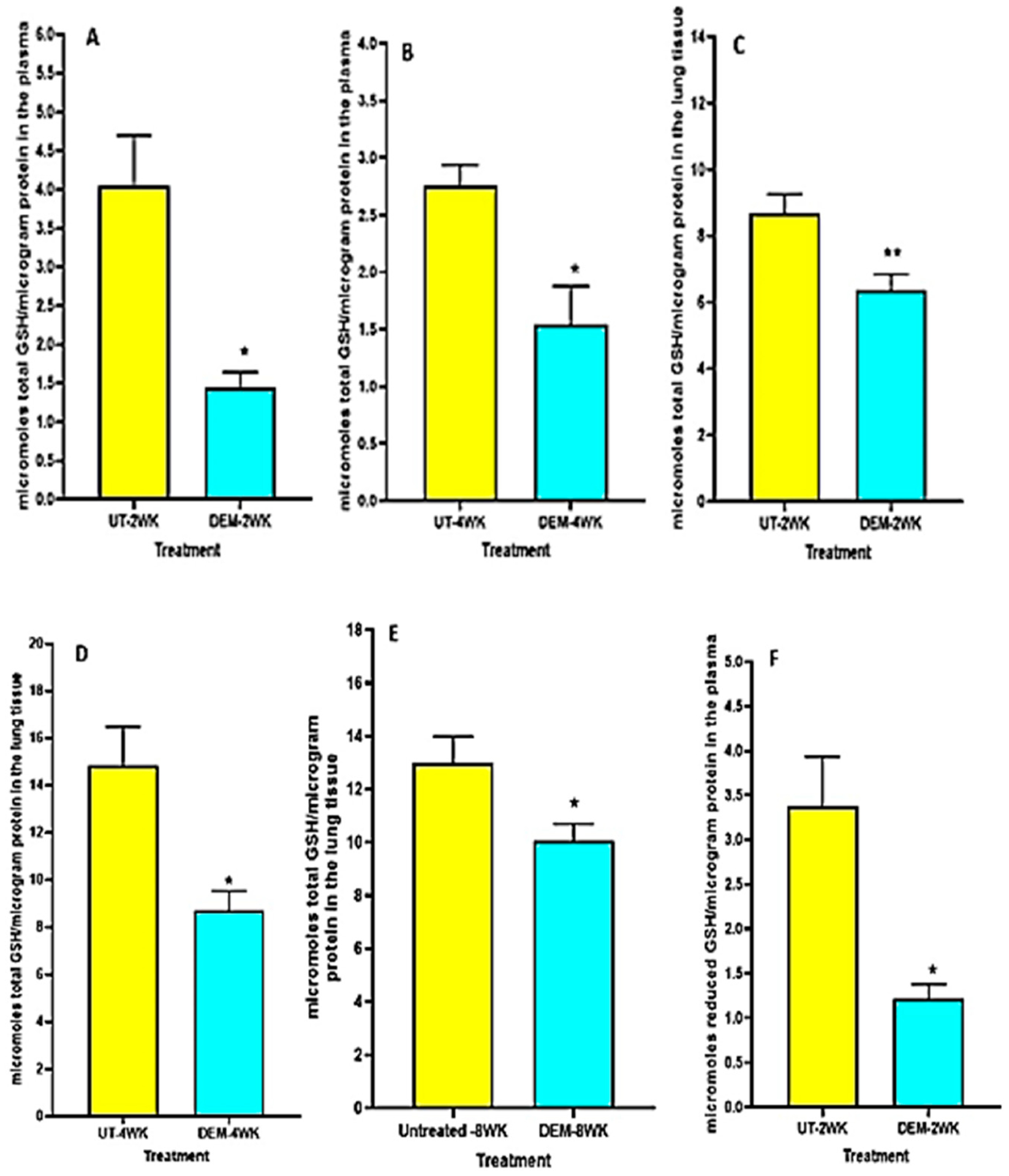

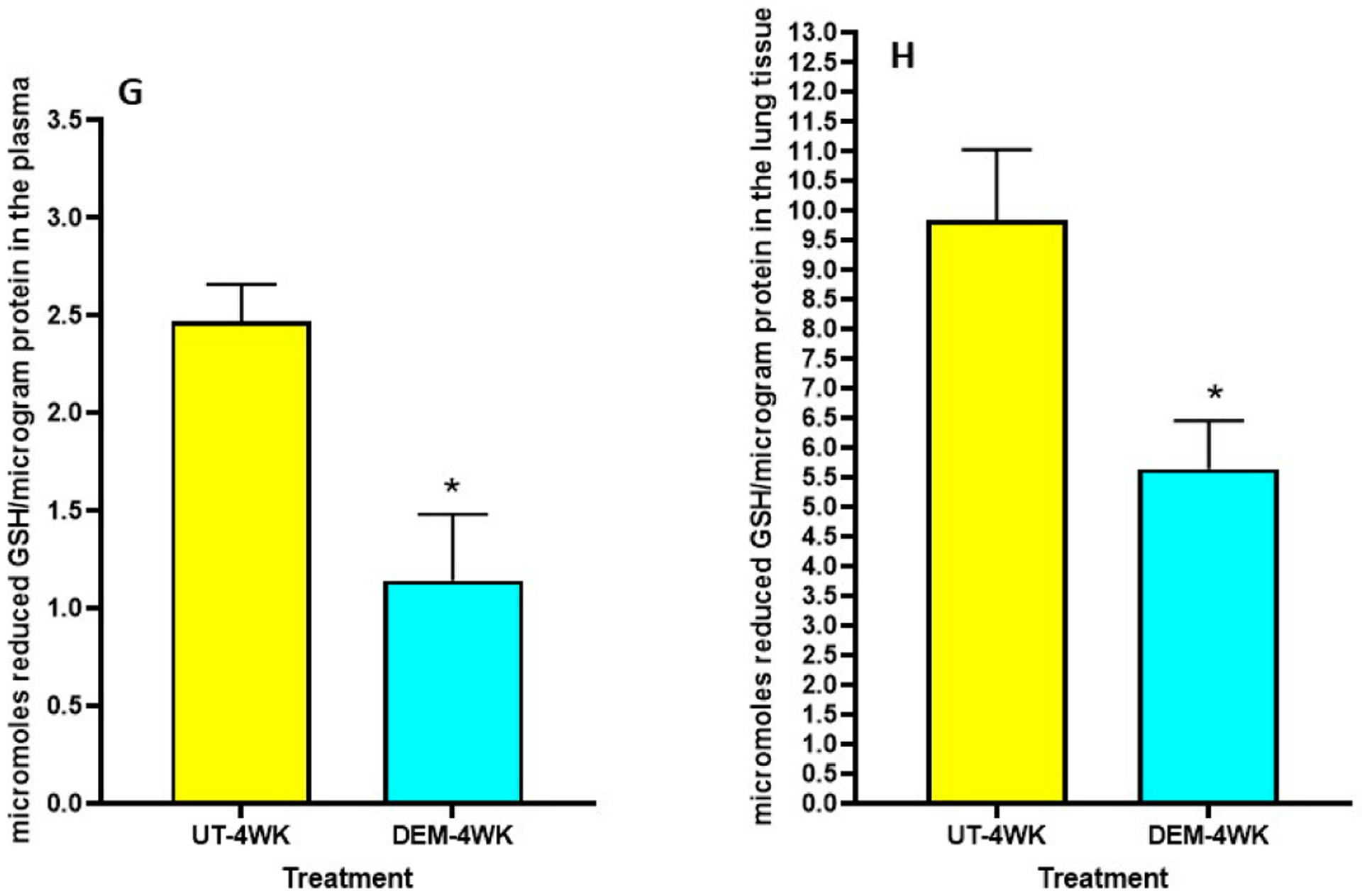
Measurement of total and reduced forms of glutathione in the lung lysates and plasma of *M. tb* infected mice that were sham-treated or treated with DEM. Total glutathione was measured in the plasma and lung lysates of *M. tb* infected mice at 2 weeks (**A**,**C**) and 4 weeks (**B**,**D**) post-infection. Reduced form of glutathione was also measured in the lung lysates and plasma of *M. tb* infected mice at 2 weeks (**E**,**F**) and 4 weeks (**G**,**H**) post-infection. Statistical analysis was performed using GraphPad Prism software. Unpaired *t* tests were performed using Welsch correction. All values reported represent the mean values for each respective category and a *p*-value of <0.05 was considered significant. Any placement of an asterisk (*) denotes a direct comparison of the DEM-treated versus untreated category. ** *p*-value < 0.005. The sample size (*n*) includes six mice each in the untreated and DEM-treated groups.

We observed a significant decrease in total GSH levels in the plasma of DEM-treated mice compared to the sham-treated control group at both 2 weeks post-infection and 4 weeks post-infection (Figure 1A,B). Furthermore, in both the 2 weeks and 4 weeks post-infection groups, the DEM-treated mice had a significant downregulation of total GSH in the lung tissue as well (Figure 1C,D). At 8 weeks post-infection, the trend continued, with a significant decrease in total GSH in the lungs for the DEM-treated mice (Figure 1E). Reduced GSH (rGSH) levels were visibly decreased in both the plasma (with a significant 50% reduction) and the lung tissue after DEM treatment in both the 2 weeks and 4 weeks post-infection groups (Figure 1F–H).

### 3.2. Assay of IL-6 and TNF-α

**Figure 2.**
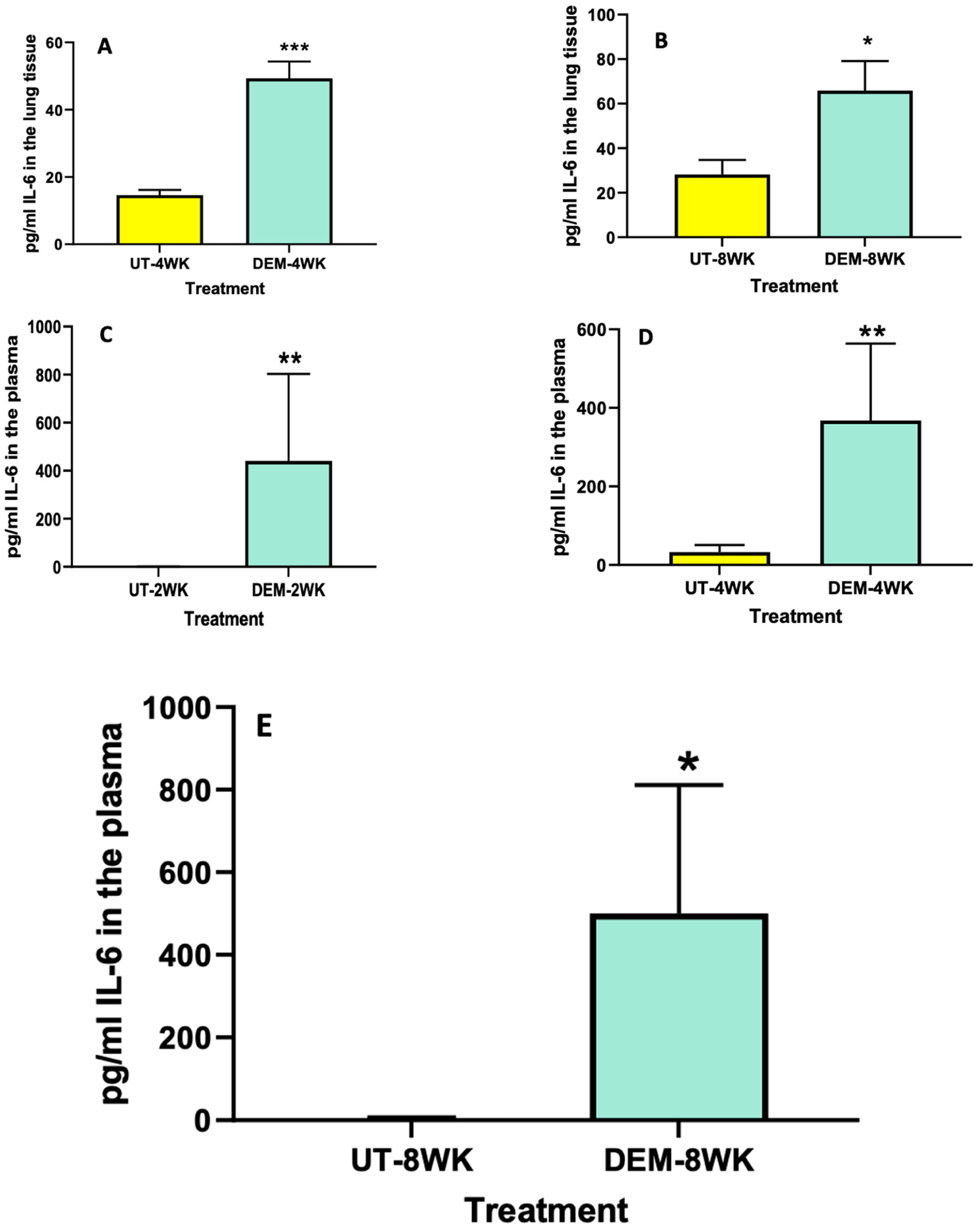
Measurement of IL-6 in the lung lysates and plasma of *M. tb* infected mice that were sham-treated or treated with DEM. IL-6 levels were measured in the lung lysates at 4 weeks (**A**) and 8 weeks (**B**) post-infection and in the plasma at 2 weeks (**C**), 4 weeks (**D**), and 8 weeks (**E**) post-*M. tb* infection. Statistical analysis was performed using GraphPad Prism software. Unpaired *t* tests were performed using Welsch correction. All values reported are representative of the mean values for each respective category and a *p*-value of <0.05 was considered significant. Any placement of an asterisk (*) denotes a direct comparison of the DEM-treated versus the untreated category. ** *p*-value < 0.005. When three asterisks are represented (***), a *p*-value below 0.0001 is implied. The sample size (*n*) includes six mice each in the untreated and DEM-treated groups.

Levels of IL-6 and TNF-α among DEM-treated mice and untreated mice were measured using ELISA. Compared to the sham group, the lung tissue of *M. tb*-infected mice treated with DEM had significantly higher levels of IL-6 at both 4 weeks (Figure 2A) and 8 weeks (Figure 2B) post-infection. Likewise, we saw a significant increase in the levels of IL-6 in the plasma of *M. tb*-infected mice treated with DEM at 2 weeks (Figure 2C), 4 weeks (Figure 2D), and 8 weeks (Figure 2E) post-infection compared to the sham group.

**Figure 3.**
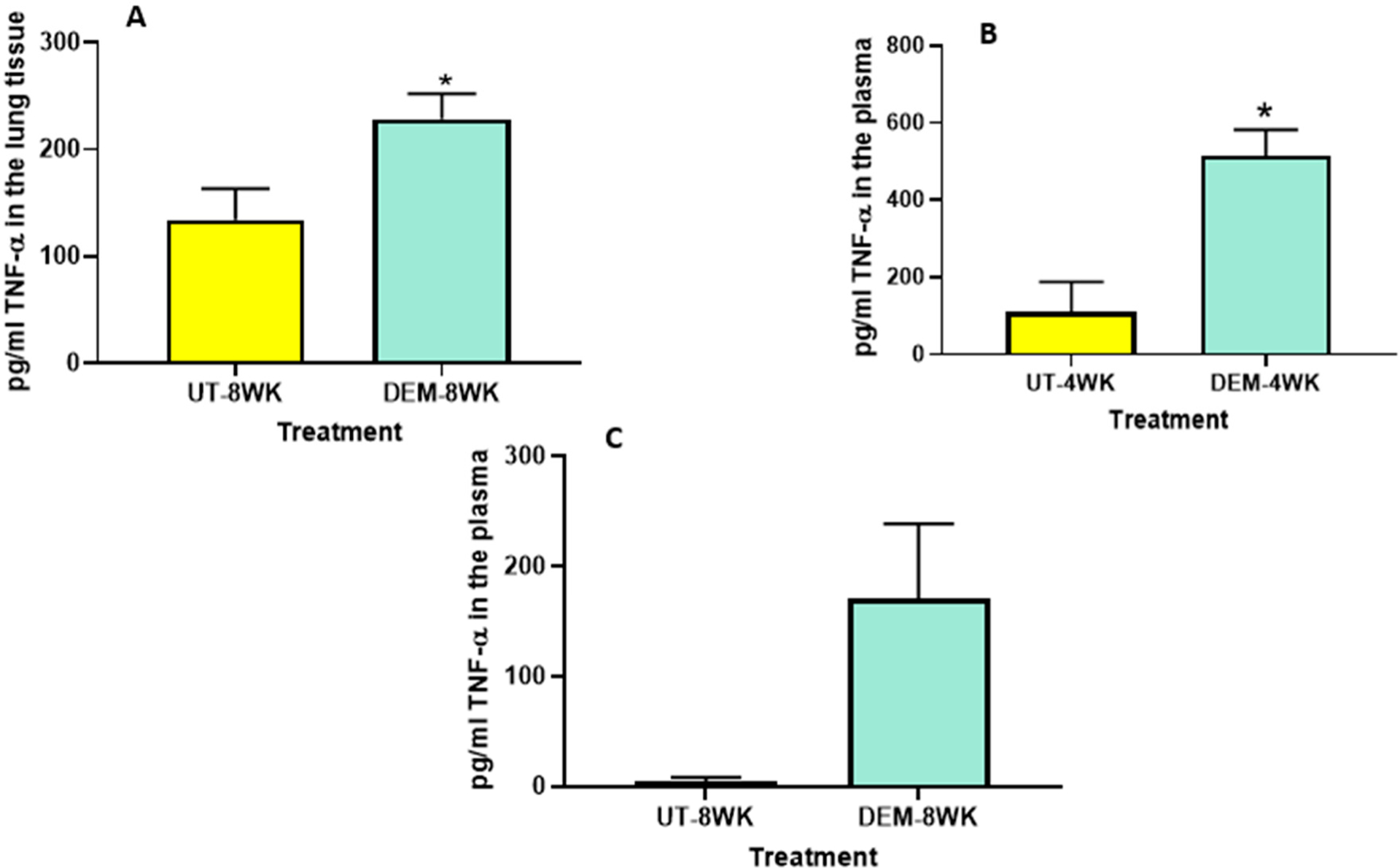
Measurement of TNF-α in the lung lysates and plasma of *M. tb* infected mice that were sham-treated or treated with DEM. Levels of TNF-α were measured in the lung lysates at 8 weeks (**A**) post-infection and in the plasma at 4 weeks (**B**) and 8 weeks (**C**) post-*M. tb* infection. Statistical analysis was performed using GraphPad Prism software. Unpaired *t* tests were performed using Welsch correction. All values reported are representative of the mean values for each respective category and a *p*-value of <0.05 was considered significant. Any placement of an asterisk (*) denotes a direct comparison of the DEM-treated versus the untreated category. The sample size (*n*) includes six mice each in the untreated and DEM-treated groups.

We also observed a significant increase in the levels of TNF-α in the lung lysates of *M. tb*-infected mice treated with DEM at 8 weeks post-infection (Figure 3A) compared to the sham group. Likewise, measurements of the plasma of *M. tb*-infected mice treated with DEM also indicated a significant increase in TNF-α levels in the 4-week (Figure 3B) group compared to the sham group. An increase was also noted in the 8-week group, although not significant (Figure 3C). These results are comparable to the IL-6 measurements and suggest the synergistic function of these cytokines in immune system regulation.

### 3.3. Assay of MDA

**Figure 4.**
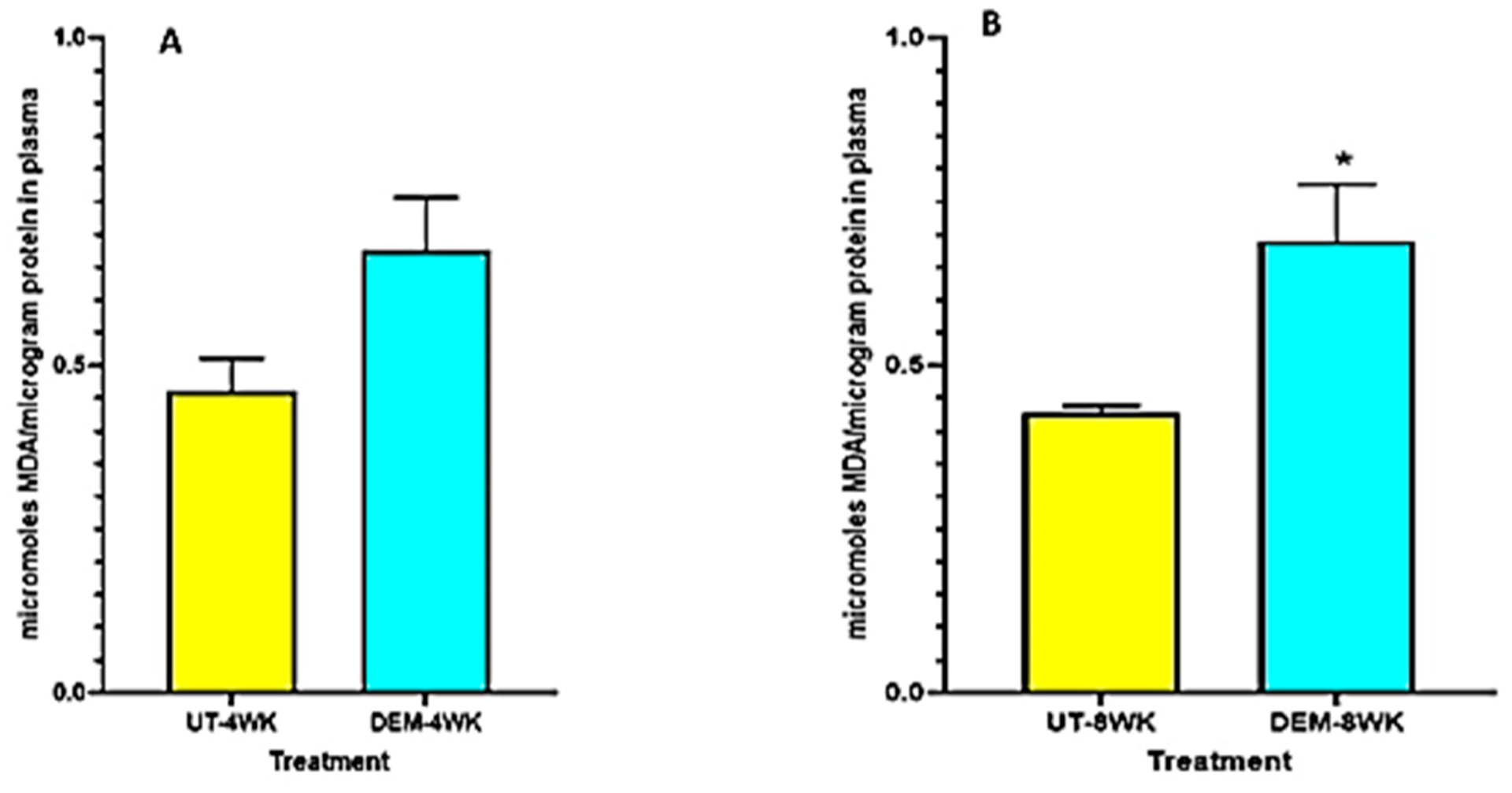
Measurement of MDA in the lung lysates and plasma of *M. tb* infected mice that were sham-treated or treated with DEM. MDA levels were measured in the plasma at 4 weeks (**A**) and 8 weeks (**B**) post-*M. tb* infection. Statistical analysis was performed using GraphPad Prism software. Unpaired *t* tests were performed using Welsch correction. All values reported are representative of the mean values for each respective category and a *p*-value of <0.05 was considered significant. Any placement of an asterisk (*) denotes a direct comparison of the DEM-treated versus the untreated category. The sample size (*n*) includes six mice each in the untreated and DEM-treated groups.

Plasma MDA levels were elevated in the DEM treatment group at 4 weeks post-infection and significantly increased at 8 weeks post-infection compared to the untreated control group (Figure 4A,B). DEM-treated mice may have experienced this increase in oxidative stress due to excess free radical production in the plasma after GSH diminishment.

### 3.4. M. tb Survival Assays in the Lungs and Spleen

**Figure 5.**
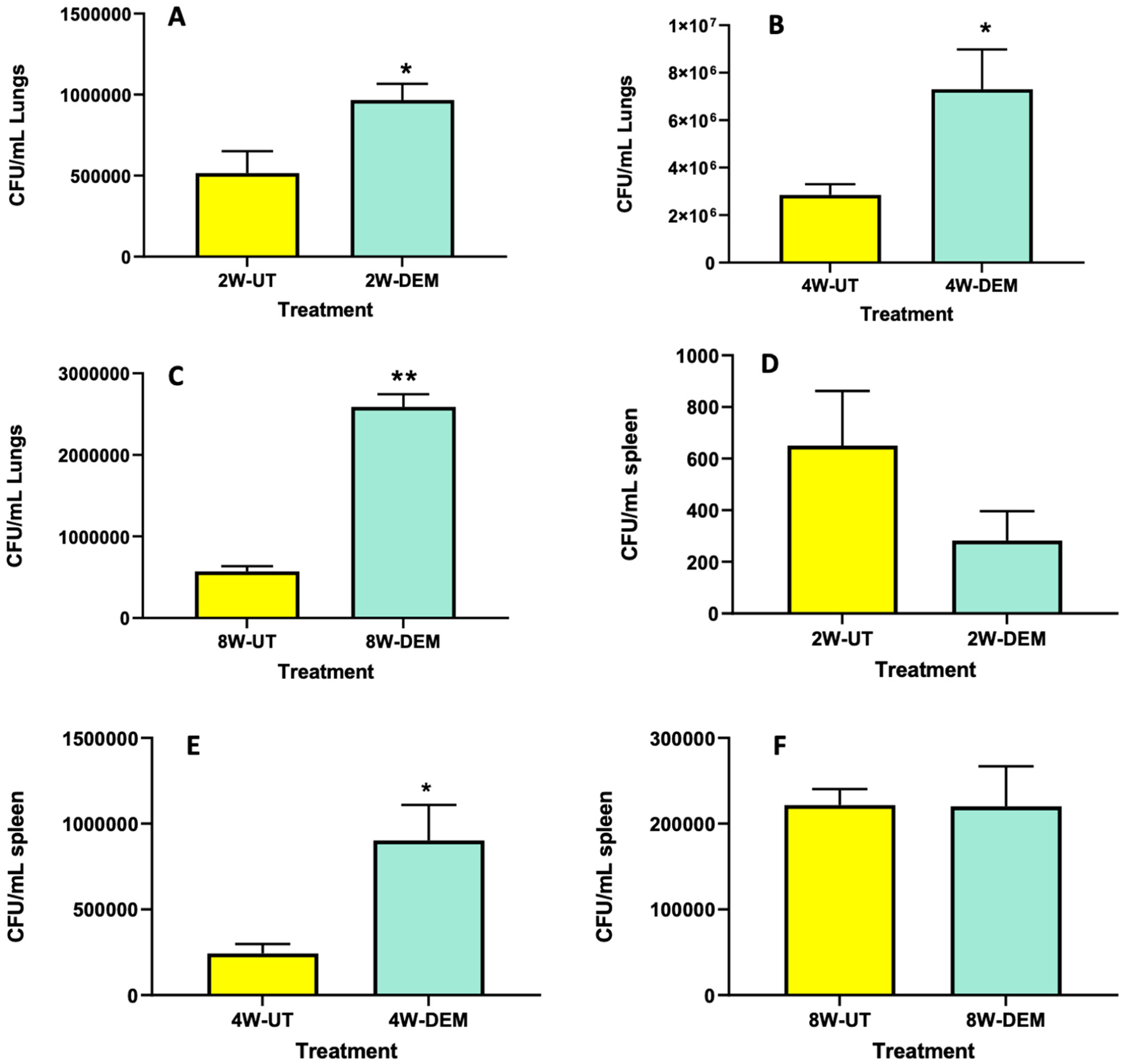
Survival of *M. tb* in the lung and spleen of mice that were sham-treated or treated with DEM. Survival of *M. tb* was determined in the lung lysates at 2 weeks (**A**), 4 weeks (**B**) and 8 weeks (**C**) post-infection and in the spleen lysates at 2 weeks (**D**), 4 weeks (**E**) and 8 weeks (**F**) post-*M. tb* infection. Statistical analysis was performed using GraphPad Prism software. Unpaired *t* tests were performed using Welsch correction. All values reported are representative of the mean values for each respective category and a p-value of <0.05 was considered significant. Any placement of an asterisk (*) denotes a direct comparison of the DEM-treated versus the untreated category. ** *p*-value < 0.005. The sample size (*n*) includes six mice each in the untreated and DEM-treated groups.

To determine overall *M. tb* survival within lung and spleen tissues, survival assays were performed. We observed that DEM treatment resulted in a significant increase in bacterial load in the lungs of mice, with a two-fold increase at 2- and 4-weeks post-infection (Figure 5A and B, respectively), as well as a four-fold increase at 8 weeks post-infection (Figure 5C). In addition, DEM treatment caused a significant four-fold increase in the survival of *M. tb* in the spleen of mice at 4 weeks post-infection (Figure 5E); however, no increase in *M. tb* survival was noted at time intervals 2- and 8-weeks post-infection (Figure 5D,F). These results indicate that GSH decrease via DEM treatment augments bacterial survival within the lung and spleen tissues and contributes to disease progression.

### 3.5. Histopathology Findings in Lungs of M. tb-Infected Mice with or without DEM Treatment

**Figure 6.**
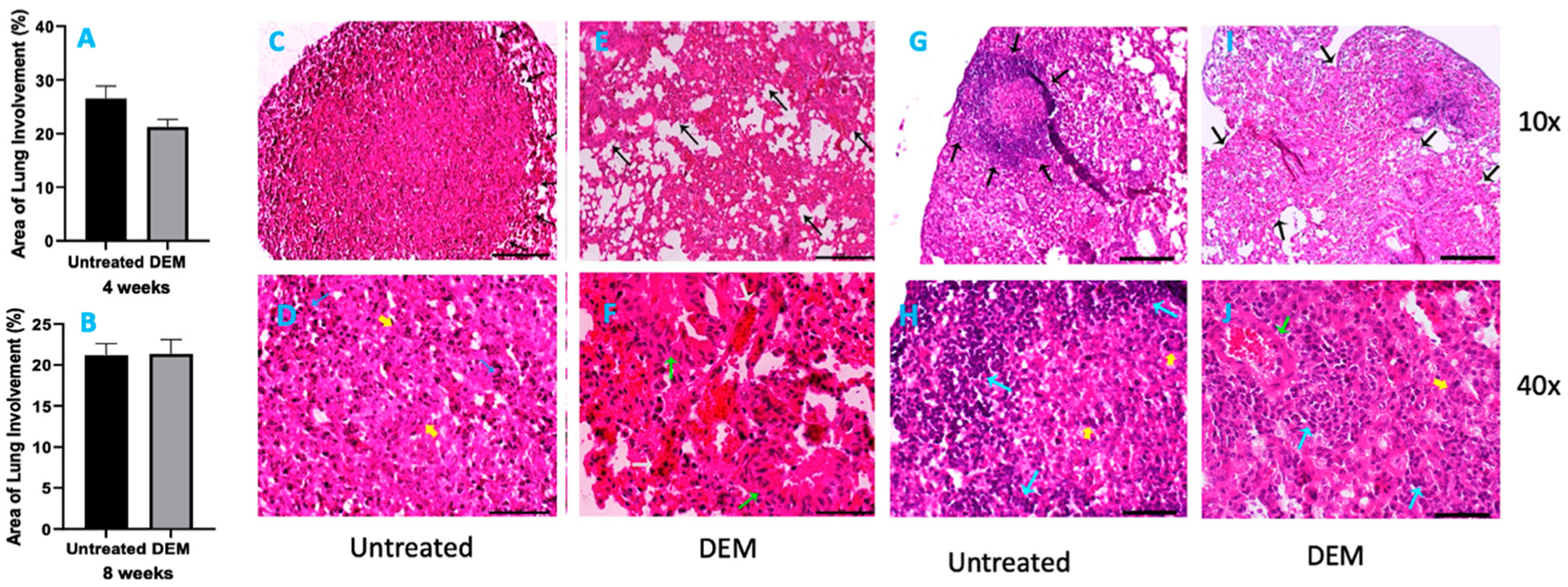
Morphometric analysis and hematoxylin and eosin staining of lung tissue sections of *M. tb*-infected mice that were sham-treated or treated with DEM. Morphometric analysis was performed in the mouse lung sections stained with hematoxylin and eosin at 4 weeks (**A**) and 8 weeks (**B**) post-*M. tb* infection. 10× (**C**,**E**,**G**,**I**) and 40× (**D**,**F**,**H**,**J**) images of hematoxylin and eosin-stained lung sections of mice at 4 weeks (**C**–**F**) and 8 weeks (**G**–**J**) post-*M. tb* infection are presented. The sample size (*n*) includes six mice each in the untreated and DEM-treated groups.

Six mice from each group were sacrificed at 2 weeks, 4 weeks (Figure 6A) and 8 weeks (Figure 6B) post-infection, and portions of lungs, liver and spleen were collected for histopathology analysis. H&E-stained tissue sections were evaluated for immune cells, and bacterial loads were calculated from the fluorescent intensity of immunohistochemically stained tissue sections. Histopathological study of lung tissue revealed large, well-formed granulomas with vast immune cell infiltration and inflammation in the *M. tb*-infected mice lung sections at 4 weeks post-infection without any treatment (Figure 6C,D). DEM-treated mice had ill-formed granulomas, reminiscent of scattered cellular aggregation in the majority of lung parenchyma (Figure 6E,F). The lungs showed vascular occlusion and bronchial congestion. Lymphocytes were found scattered in the highly cellular areas. No visible necrosis of the lesion was noted (Figure 6E,F). The architecture of lung granulomas in *M. tb*-infected mice at 8 weeks post-infection resembled the architecture observed at 4 weeks. However, untreated mice had lymphocyte-rich cuffs surrounding the central core of the granulomas (Figure 6G,H). In contrast, the DEM-treated mice at 8 weeks post-infection had much bigger areas of immune cell infiltration and more congestion of bronchial, as well as vascular, structures (Figure 6I,J) compared to 4 weeks post-infection. GSH deficiency significantly increased the pulmonary and extrapulmonary bacterial load.

## 4. Discussion

DEM-treated mice serve as models which allow us to study the effects of GSH diminishment (such as those induced by T2DM or other causes) on TB patients. Furthermore, treating mice with the GSH-decreasing agent, DEM, while comparing them to untreated mice substantiates the positive effects of GSH supplementation observed in our previous studies. DEM has been known to reduce GSH levels for many years. A study by Mitchell et al. suggests that DEM depletes cellular GSH to less than 5% of the GSH in untreated cells [26], deeming DEM a proper agent to study the effects of depleting GSH levels.

The treatment of *M. tb*-infected C57BL/6 (WT) mice with DEM (50 mM) led to effects similar to those seen in individuals with low GSH levels [8,13]. For example, the DEM-treated mice had significantly increased levels of proinflammatory cytokines, including IL-6, in the lung and plasma (Figure 2). We also observed increased TNF-α levels in the lungs and plasma of mice (Figure 3). Furthermore, treatment with DEM led to an increase in reactive oxygen species (ROS) in the lung and plasma of the mice (Figure 4), confirming that rGSH levels have been depleted. In fact, our results show that rGSH and total GSH levels were downregulated by 50% in the plasma and in the lungs of mice treated with DEM for 2 and 4 weeks (Figure 1). Total GSH was also significantly diminished in the lung tissue of mice treated with DEM at 2 weeks post-infection. Such a significant decrease in rGSH levels was also observed by Kaur et al. in BAL/c mice treated with DEM [27].

This further demonstrates that the depletion of GSH leads to an increase in oxidative stress. This failed redox response and exacerbated inflammatory response induce an increased survival of *M. tb*. The significantly increased survival of *M. tb* in both the lungs and the spleen of the DEM-treated mice suggests the importance of GSH in controlling *M. tb* infection (Figure 5). GSH supplementation has been previously shown to significantly decrease *M. tb* survival in the PBMCs of people infected with HIV and in human monocyte-derived macrophages of people with T2DM [8,13]. *M. tb* survival in the mice samples also increased in a dose-dependent manner, with pathogen count increasing with an increase in DEM concentration (Figure 5). These observations suggest poor mycobacterial control in DEM-treated mice, which is typically controlled by the granuloma.

Thus, it is important to elucidate granuloma formation in the lungs of DEM-treated mice. A granuloma is an organized aggregation of immune cells that forms in response to a repeated stimulus; it is also known as a small area of inflammation. Current literature acknowledges that *M. tb* infection is the most common cause of granuloma formation [4]. The purpose of granuloma formation during *M. tb* infection is to diminish the growth of the pathogen by forming aggregates of infected cells. Not surprisingly, the granulomas of DEM-treated mice were poorly formed, and the aggregates were relatively scattered, especially at 8 weeks post-infection (Figure 6). These mice had much larger areas of immune cell infiltration and more congestion of bronchial and vascular structures than the 4 weeks post-infection control group.

Limitations of our study include using a mouse model of TB, which may lead to minor differences in results compared to humans. In further studies, our lab plans to treat diabetic mice (infected with *M. tb*) with lGSH and DEM to better understand the effects of GSH supplementation with TB and T2DM comorbidity.

## Data Availability

The data presented in this study are available on request from the corresponding author.

## References

[1] World Health Organization. Global Tuberculosis Report 2020; World Health Organization: Geneva, Switzerland, 2020.

[2] Pai M; Behr MA; Dowdy D; Dheda K; Divangahi M; Boehme CC; Ginsberg A; Swaminathan S; Spigelman M; Getahun H; Tuberculosis. Nat. Rev. Dis. Primers 2016, 2, 16076. 27784885 10.1038/nrdp.2016.76

[3] Houben D; Demangel C; Van Ingen J; Perez-Galarza J; Baldeón L; Abdallah AM; Caleechurn L; Bottai D; Van Zon M; De Punder K; ESX-1-mediated translocation to the cytosol controls virulence of mycobacteria. Cell. Microbiol 2012, 14, 1287–1298. 22524898 10.1111/j.1462-5822.2012.01799.x

[4] Ramakrishnan L Revisiting the role of the granuloma in tuberculosis. Nat. Rev. Immunol 2012, 12, 352–366. 22517424 10.1038/nri3211

[5] Guirado E; Mbawuike U; Keiser TL; Arcos J; Azad AK; Wang S-H; Schlesinger LS Characterization of Host and Microbial Determinants in Individuals with Latent Tuberculosis Infection Using a Human Granuloma Model. mBio 2015, 6, e02537–14. 25691598 10.1128/mBio.02537-14 PMC4337582

[6] Ferlita S; Yegiazaryan A; Noori N; Lal G; Nguyen T; To K; Venketaraman V Type 2 Diabetes Mellitus and Altered Immune System Leading to Susceptibility to Pathogens, Especially Mycobacterium tuberculosis. J. Clin. Med 2019, 8, 2219. 10.3390/jcm8122219 PMC6947370 31888124

[7] Dutta NK; Karakousis PC Latent Tuberculosis Infection: Myths, Models, and Molecular Mechanisms. Microbiol. Mol. Biol. Rev 2014, 78, 343–371. 25184558 10.1128/MMBR.00010-14 PMC4187682

[8] Lagman M; Ly J; Saing T; Singh MK; Tudela EV; Morris D; Chi P-T; Ochoa C; Sathananthan A; Venketaraman V Investigating the Causes for Decreased Levels of Glutathione in Individuals with Type II Diabetes. PLoS ONE 2015, 10, e0118436. 25790445 10.1371/journal.pone.0118436 PMC4366217

[9] Guerra C; Johal K; Morris D; Moreno S; Alvarado O; Gray D; Tanzil M; Pearce D; Venketaraman V Control of Mycobacterium tuberculosis growth by activated natural killer cells. Clin. Exp. Immunol 2012, 168, 142–152. 22385249 10.1111/j.1365-2249.2011.04552.x PMC3390505

[10] Guerra C; Morris D; Sipin A; Kung S; Franklin M; Gray D; Tanzil M; Guilford F; Khasawneh FT; Venketaraman V Glutathione and Adaptive Immune Responses against Mycobacterium tuberculosis Infection in Healthy and HIV Infected Individuals. PLoS ONE 2011, 6, e28378. 22164280 10.1371/journal.pone.0028378 PMC3229597

[11] Ly J; Lagman M; Saing T; Singh MK; Tudela EV; Morris D; Anderson J; Daliva J; Ochoa C; Patel N; Liposomal Glutathione Supplementation Restores TH1 Cytokine Response to Mycobacterium tuberculosis Infection in HIV-Infected Individuals. J. Interf. Cytokine Res 2015, 35, 875–887. 10.1089/jir.2014.0210 PMC4642835 26133750

[12] Hunter G; Eagles BA Glutathione: A critical study. J. Biol. Chem 1927, 72, 147–166.

[13] Teskey G; Abrahem R; Cao R; Gyurjian K; Islamoglu H; Lucero M; Martinez A; Paredes E; Salaiz O; Robinson B; Glutathione as a Marker for Human Disease. Adv. Clin. Chem 2018, 87, 141–159. 30342710 10.1016/bs.acc.2018.07.004

[14] Venketaraman V; Dayaram YK; Talaue MT; Connell ND Glutathione and Nitrosoglutathione in Macrophage Defense against Mycobacterium tuberculosis. Infect. Immun 2005, 73, 1886–1889. 15731094 10.1128/IAI.73.3.1886-1889.2005 PMC1064956

[15] Teskey G; Cao R; Islamoglu H; Medina A; Prasad C; Prasad R; Sathananthan A; Fraix M; Subbian S; Zhong L; The Synergistic Effects of the Glutathione Precursor, NAC and First-Line Antibiotics in the Granulomatous Response against Mycobacterium tuberculosis. Front. Immunol 2018, 9, 2069. 30258443 10.3389/fimmu.2018.02069 PMC6144952

[16] Sutherland APR; Wali J; Thomas H Linking obesity with type 2 diabetes: The role of T-bet. Diabetes Metab. Syndr. Obes. Targets Ther 2014, 7, 331–340. 10.2147/DMSO.S51432 PMC4113403 25092994

[17] To K; Cao R; Yegiazaryan A; Owens J; Nguyen T; Sasaninia K; Vaughn C; Singh M; Truong E; Medina A; Effects of Oral Liposomal Glutathione in Altering the Immune Responses Against Mycobacterium tuberculosis and the Mycobacterium bovis BCG Strain in Individuals With Type 2 Diabetes. Front. Cell. Infect. Microbiol 2021, 11, 468. 10.3389/fcimb.2021.657775 PMC8211104 34150674

[18] Islamoglu H; Cao R; Teskey G; Gyurjian K; Lucar S; Fraix MP; Sathananthan A; Chan JK; Venketaraman V Effects of ReadiSorb L-GSH in Altering Granulomatous Responses against Mycobacterium tuberculosis Infection. J. Clin. Med 2018, 7, 40. 10.3390/jcm7030040 PMC5867566 29494546

[19] To K; Cao R; Yegiazaryan A; Owens J; Sasaninia K; Vaughn C; Singh M; Truong E; Sathananthan A; Venketaraman V The Effects of Oral Liposomal Glutathione and In Vitro Everolimus in Altering the Immune Responses against Mycobacterium bovis BCG Strain in Individuals with Type 2 Diabetes. Biomol. Concepts 2021, 12, 16–26. 33966361 10.1515/bmc-2021-0003 PMC8975622

[20] Costa LG; Murphy SD Effect of diethylmaleate and other glutathione depletors on protein synthesis. Biochem. Pharmacol 1986, 35, 3383–3388. 3768026 10.1016/0006-2952(86)90439-9

[21] Kumar SM; Dey A Regulation of Glutathione in Health and Disease with Special Emphasis on Chronic Alcoholism and Hyperglycaemia Mediated Liver Injury: A Brief Perspective. Springer Sci. Rev 2014, 2, 1–13.

[22] David V; Bachelez H; Leca G; Degos L; Boumsell L; Bensussan A Identification of a novel 110-kilodalton structure expressed on a subset of T cell receptor-gamma delta-bearing cloned lymphocytes. J. Immunol 1990, 144, 1–6. 2136878

[23] Subbian S; Pandey R; Soteropoulos P; Rodriguez GM Vaccination with an Attenuated Ferritin Mutant Protects Mice against VirulentMycobacterium tuberculosis. J. Immunol. Res 2015, 2015, 385402. 26339659 10.1155/2015/385402 PMC4539171

[24] Chatterjee S; Khunti K; Davies MJ Type 2 diabetes. Lancet 2017, 389, 2239–2251. 28190580 10.1016/S0140-6736(17)30058-2

[25] Franklin CC; Rosenfeld-Franklin ME; White C; Kavanagh TJ; Fausto N TGFβ1-induced suppression of glutathione antioxidant defenses in hepatocytes: Caspase-dependent posttranslational and caspase-independent transcrip-tional regulatory mechanisms. FASEB J. 2003, 17, 1–23. 12824300 10.1096/fj.02-0867fje

[26] Kaur P; Kalia S; Bansal MP Effect of Diethyl Maleate Induced Oxidative Stress on Male Reproductive Activity in Mice: Redox Active Enzymes and Transcription Factors Expression. Mol. Cell. Biochem 2006, 291, 55–61. 16941228 10.1007/s11010-006-9195-6

[27] Sugawara I; Mizuno S; Yamada H; Matsumoto M; Akira S Disruption of Nuclear Factor-Interleukin-6, a Transcription Factor, Results in Severe Mycobacterial Infection. Am. J. Pathol 2001, 158, 361–366. 11159172 10.1016/S0002-9440(10)63977-6 PMC1850332

[28] Tsenova L; Fallows D; Kolloli A; Singh P; O’Brien P; Kushner N; Kaplan G; Subbian S Inoculum size and traits of the infecting clinical strain define the protection level against Mycobacterium tuberculosis infection in a rabbit model. Eur. J. Immunol 2020, 50, 858–872. 32130727 10.1002/eji.201948448

